# Silencing Bcl-2 Expression in Epithelial Cancer Cells Using “Smart” Particles

**DOI:** 10.3390/jfb5030167

**Published:** 2014-09-16

**Authors:** Yen-Ling Lin, Guohua Jiang, Zhaocheng Zhang, Jacques E. Nör, Mohamed E. H. ElSayed

**Affiliations:** 1 Department of Biomedical Engineering, University of Michigan, 1101 Beal Ave., Ann Arbor, MI 48109, USA; E-Mail: yenling225@gmail.com (Y.-L.L.); 2 Department of Cariology, Restorative Sciences, and Endodontics, University of Michigan, 1011 N. University Ave., Ann Arbor, MI 48109, USA; E-Mails: zczhang@umich.edu (Z.Z.); jenor@umich.edu (J.E.N.); 3 Macromolecular Science and Engineering Program, University of Michigan, 2300 Hayward Ave., Ann Arbor, MI 48109, USA

**Keywords:** pH-sensitive comb-like polymers, smart particles, endosomal escape, cytoplasmic siRNA delivery, Bcl-2 knockdown

## Abstract

Short interfering RNA (siRNA) targeted against anti-apoptotic Bcl-2 protein proved to knockdown its expression and trigger cancer cell death. We used degradable, pH-sensitive, comb-like [P(EAA-co-BMA)-b-PNASI-g-P(HMA-co-TMAEMA)] polymer to condense anti-Bcl-2 siRNA into “smart” particles, which proved to shuttle their cargo past the endosomal membrane and into the cytoplasm of HeLa and UM-SCC-17B cancer cells. HeLa and UM-SCC-17B cancer cells were treated with anti-Bcl-2 particles followed by quantifying Bcl-2 mRNA and protein levels using qRT-PCR and western blotting, respectively. “Smart” anti-Bcl-2 particles selectively suppress Bcl-2 mRNA and protein levels in HeLa cells by 50%–60% and 79%–81%, respectively. Similarly, “smart” anti-Bcl-2 particles inhibited Bcl-2 mRNA levels by 30%, 40%, and 20% upon incubation with UM-SCC-17B cancer cells for 48, 72, and 96 h, respectively. Bcl-2 protein expression in UM-SCC-17B cancer cells was inhibited by 30% after treatment for 72 h. Results show that pH-sensitive comb-like polymer complex anti-Bcl-2 siRNA forming “smart” nanoparticles that deliver their cargo into the cytoplasm of HeLa and UM-SCC-17B cancer cells causing Bcl-2 knockdown at the mRNA and protein levels.

## 1. Introduction

B-cell lymphoma 2 (Bcl-2) is a family of proteins that includes more than 20 apoptotic regulators with opposing functions but share at least one conserved Bcl-2 homology (BH) domain [1,2,3]. Anti-apoptotic proteins such as Bcl-2, Bcl-X_L_, and Bcl-w appear to inhibit apoptotic cell death through their binding to the pro-apoptotic proteins [1,2,3]. Pro-apoptotic proteins are sub-grouped into the “Bax” family proteins, which have several domains that are homologous to the domains of anti-apoptotic proteins [1,2,3]. The “BH3-only” family proteins have the BH3 domain that is conserved in anti-apoptotic proteins [1,2,3]. In response to the death signal, “Bax” family proteins such as Bax and Bak form homo-oligomers on the mitochondrial membrane, which result in the cytoplasmic release of cytochrome c and initiating the caspase cascade that eventually leads to apoptotic cell death [1,2,3]. In comparison, Bcl-2 is a pro-survival protein that is over-expressed in multiple human cancer cells including head and neck cancer, which prevents cancer cell death [4,5].

The anti-apoptotic activity of Bcl-2 protein is attributed to its ability to stabilize the mitochondrial membrane and inhibit the cytoplasmic release of cytochrome c, which prevents the activation of caspases and initiation of cell apoptosis [5,6]. Overexpression of anti-apoptotic Bcl-2 protein in head and neck cancer cells has been linked to increased resistance to radio- and chemotherapy and is considered a viable therapeutic target [7,8]. Antisense oligodeoxynucleotides (ASODN) and short interfering RNA (siRNA) molecules have been used to silence the expression of anti-apoptotic Bcl-2 protein in head and neck cancer cells, which proved to successfully induce cancer cell death in response to chemotherapy both *in vitro* and *in vivo* [9,10]. However, transforming ASODN and siRNA molecules into effective therapies remains a significant challenge due to the lack of efficient and biocompatible carriers that can shuttle a large dose of the nucleic acid drug past the endosome membrane and into the cytoplasm of targeted cancer cells.

We reported the design and synthesis of a new degradable, comb-like, pH-sensitive, and membrane-destabilizing polymer namely P(EAA-*co*-BMA)-*b*-PNASI-*g*-P(HMA-*co*-TMAEMA), which incorporates two blocks in the backbone [11]. The first block is composed of pH-sensitive ethyl acrylic acid (EAA) monomers and hydrophobic butyl methacrylate (BMA) monomers whereas the second block incorporates *N*-acryloxy succinimide (NASI) monomers, which allows controlled grafting of hydrophobic hexyl methacrylate (HMA) and cationic *N*,*N*,*N*-trimethyl aminoethyl methacrylate (TMAEMA) copolymers via acid-labile hydrazone linkages (Scheme IA). At physiologic pH, this polymer retains its comb-like architecture and successfully complex siRNA molecules through electrostatic interactions with the cationic grafts forming “smart” pH-sensitive particles (Scheme IB) that are taken up into the cell by adsorptive endocytosis (Scheme IC). In the endosome, the comb-like polymer “senses” the drop in environment pH, which results in hydrolysis of the acid-labile hydrazone linkage and release of the hydrophobic/cationic grafts that rupture the endosomal membrane and release the siRNA cargo into the cytoplasm (Scheme ID). We report the ability of P(EAA-*co*-BMA)-*b*-PNASI-*g*-P(HMA-*co*-TMAEMA) polymer to condense anti-Bcl-2 siRNA into “smart” particles that deliver their cargo past the endosomal membrane and into the cytoplasm of HeLa cervical cancer and UM-SCC-17B head and neck cancer cells to knockdown the expression of anti-apoptotic Bcl-2 protein at the mRNA and protein levels.

**Scheme I:**
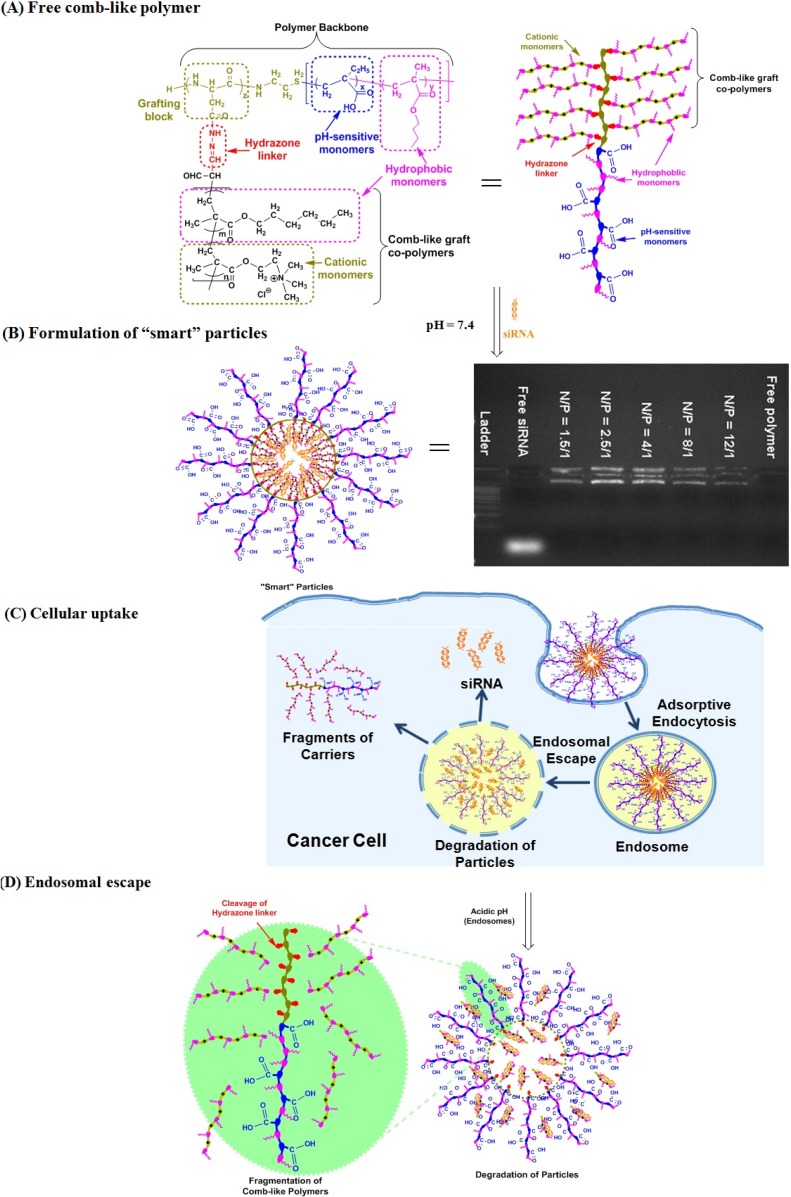
A schematic drawing showing (**A**) the chemical structure of a degradable, pH-sensitive, membrane-destabilizing, comb-like polymer. The first block in the diblock polymer backbone incorporates pH-sensitive EAA and hydrophobic BMA monomers. The second block incorporates *N*-acryloxy succinimide (NASI) monomers, which are functionalized to allow controlled grafting of hydrophobic HMA and cationic TMAEMA monomers via acid-labile hydrazone linkages. Comb-like polymer (**A**) condenses siRNA molecules forming “smart” particles (**B**); after internalization into target cells through adsorptive endocytosis (**C**); the acid-labile hydrazone linkages get hydrolysed releasing the membrane-active fragments that rupture the endosomal membrane (**D**) and release the encapsulated cargo into the cytoplasm. Image in panel B (right) shows a 1% w/V agarose gel stained with ethidium bromide to visualize the electrophoretic mobility of free siRNA and the particles prepared by complexing P(EAA-*co*-BMA)-*b*-PNASI-*g*-P(HMA-*co*-TMAEMA) comb-like polymer with 0.7 µg of anti-Bcl-2 siRNA at different N/P (+/−) ratios.

## 2. Results and Discussion

### 2.1. Formulation and Characterization of “Smart” Particles

The ability of P(EAA-*co*-BMA)-*b*-PNASI-*g*-P(HMA-*co*-TMAEMA)comb-like polymer to condense anti-Bcl-2 siRNA molecules into pH-sensitive particles was analyzed using the standard gel retardation assay. Comb-like polymer was mixed with a fixed amount (0.7 µg) of anti-Bcl-2 siRNA molecules at different N/P (+/−) ratios. The loaded RNA molecules were encapsulated into stable particles as a result of the electrostatic interaction between the cationic quaternary amine (N/+) groups of the TMAEMA monomers and the anionic phosphate (P/−) groups of the RNA molecules. Results showed that P(EAA-*co*-BMA)-*b*-PNASI-*g*-P(HMA-*co*-TMAEMA) comb-like polymer successfully complexed the loaded siRNA molecules at all N/P ratios, which is indicated by their retention in the loading wells, while free siRNA molecules migrate towards the positive electrode (Scheme IB).

**Figure 1:**
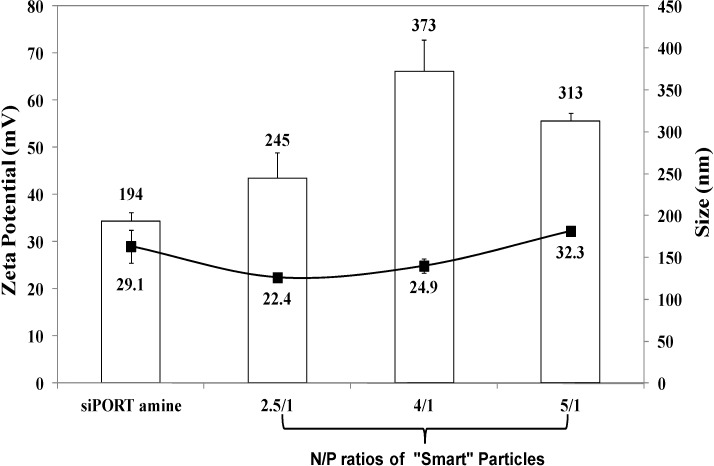
The size and zeta potential of siPORT amine-based complexes and “smart” particles prepared by complexation of P(EAA-*co*-BMA)-*b*-PNASI-*g*-P(HMA-*co*-TMAEMA) comb-like polymer with 1.14 µg of anti-GAPDH siRNA at N/P (+/−) ratios of 2.5/1, 4/1, and 5/1. The plotted results are the average ± the standard error of the mean of two independent experiments each carried out in triplicates.

Results show that the polymer can condense siRNA molecules at lower N/P ratios compared to those of other acrylic acid-based polymers [12,13], thus reducing the amount of comb-like polymer needed to complex a given dose of therapeutic nucleic acids and consequently minimizing the toxicity commonly associated with excess cationic carriers [14,15]. Size and surface charge of the particles prepared at N/P ratios of 2.5/1, 4/1, and 5/1 were measured using dynamic light scattering and zeta potential measurements, respectively. Results show that particles prepared at N/P ratios of 2.5/1, 4/1, and 5/1 have an average size of 245, 373, and 313 nm, respectively (Figure 1). These particles carry positive surface charges of 22.4, 24.9, and 32.3 mV at N/P ratios of 2.5/1, 4/1, and 5/1, respectively. siPORT amine, which is a commercial polymer-based transfection reagent was used a positive control in our studies. Results show that the size of “smart” particles is slightly larger than siPORT amine-based complexes, while the surface charges are relatively similar. The size of “smart” particles proved to fall below the size cut off size of 400–600 nm for tumor vasculature [16], which when coupled with their cationic nature will facilitate particle’s interaction and internalization into target cells via adsorptive endocytosis.

### 2.2. Uptake of “Smart” Particles by HeLa and UM-SCC-17B Cells

**Figure 2:**
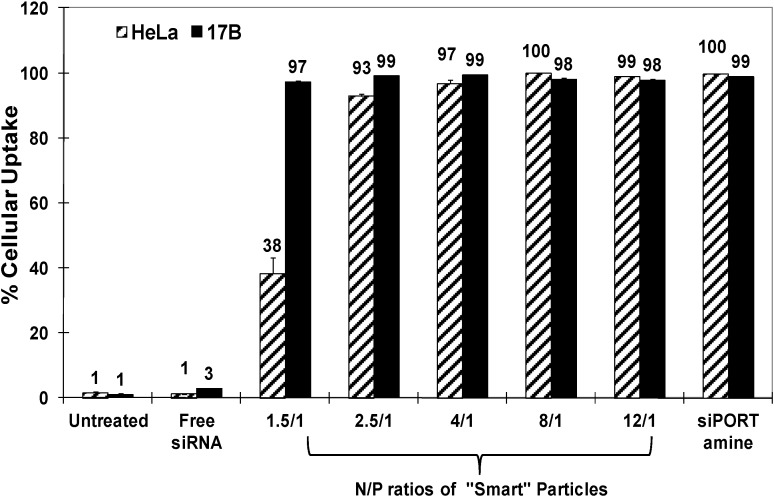
Percentage of HeLa and UM-SCC-17B cancer cells that internalize siPORT amine-based complexes and “smart” nanoparticles prepared by complexation of P(EAA-*co*-BMA)-*b*-PNASI-*g*-P(HMA-*co*-TMAEMA) comb-like polymer with 1.14 µg of fluorescently-labeled anti-GAPDH siRNA at different N/P (+/−) ratios upon incubation for 6 h in a serum-free culture medium. Results are the average + the standard error of the mean of three replicates.

We evaluated the internalization of fluorescently-labeled “smart” particles prepared at different N/P ratios into HeLa cervical carcinoma and UM-SCC-17B head and neck squamous cell carcinoma using flow cytometry. Complexes prepared using commercial siPORT amine transfection agents were used as positive controls. Figure 2 shows that “smart” particles are efficiently (>97%) taken up into HeLa and UM-SCC-17B cells at N/P ratios higher than 2.5/1, and siPORT amine-based complexes also showed high internalization (~100%) into both cell types. The relatively lower uptake into HeLa cells using particles prepared at N/P of 1.5/1 could be due to the different cell membrane compositions between HeLa and UM-SCC-17B cancer cells [17]. These results indicate that our “smart” particles can be successfully internalized by HeLa and UM-SCC-17B cancer cells through adsorptive endocytosis due to the positive surface charge of these particles. Earlier research showed that the increase of particle’s positive surface charge is typically associated with toxicity or low transfection efficiency due to poor decomplexation of the loaded DNA/RNA molecules [18,19,20]. Consequently, we decided to evaluate the transfection efficiency of the particles prepared at N/P ratios of 2.5/1, 4/1 and 6/1, which will have a sufficient number of cationic TMAEMA residues to complex the loaded siRNA molecules, while eliminating cellular toxicity without preventing cytoplasmic decomplexation of the loaded siRNA molecules.

### 2.3. Effect of “Smart” Particles on GAPDH Expression

**Figure 3:**
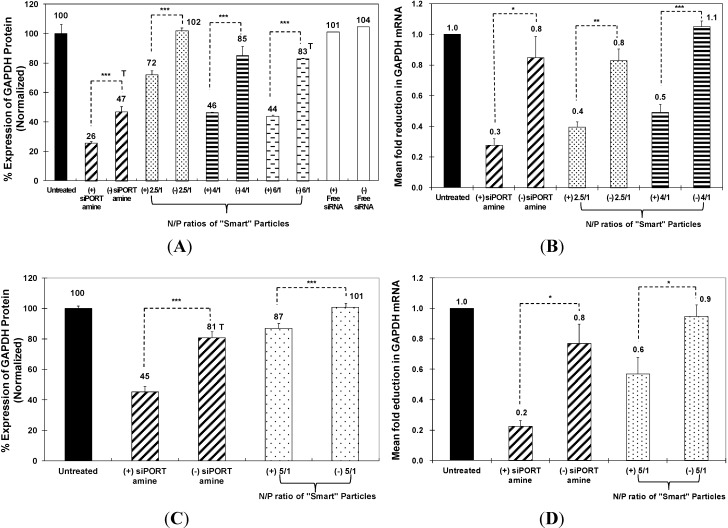
Effect of siPORT amine-based complexes and “smart” nanoparticles prepared by complexation of P(EAA-*co*-BMA)-*b*-PNASI-*g*-P(HMA-*co*-TMAEMA) comb-like polymer with 1.14 µg of the anti-GAPDH siRNA (+) or scrambled siRNA (−) at N/P (+/−) ratios of 2.5/1, 4/1, and 6/1 (**A**,**B**) or 5/1 (**C**,**D**) on GAPDH protein (**A**,**C**) and mRNA levels (**B**,**D**) in HeLa cervical cancer cells (**A**,**B**) and in UM-SCC-17B head and neck cancer cells (C,D). Levels of GAPDH mRNA are normalized to the levels of β-actin. Results are the average + the standard error of the mean of five replicates. Statistical difference between particles encapsulating anti-GAPDH siRNA (+) and scrambled siRNA (−) was evaluated using paired t test where * denotes *p* ≤ 0.05, ** denotes *p* ≤ 0.01, and *** denotes *p* ≤ 0.005.

The ability of “smart” particles to achieve functional delivery of complexed siRNA molecules into the cytoplasm of HeLa and UM-SCC-17B cancer cells was evaluated based on their ability to selectively knockdown GAPDH gene expression at the mRNA and protein levels. We utilized the KDalert GAPDH assay kit to measure the changes in GAPDH protein level upon incubation with particles that encapsulate the anti-GAPDH siRNA molecules. These particles were compared to those encapsulating a scrambled siRNA sequence. We utilized siPORT amine-based complexes encapsulating an equal dose of anti-GAPDH siRNA molecules as a positive control to determine the maximum level of knockdown that can be achieved using robust commercial transfection agents. As shown in Figure 3A, particles prepared at N/P ratios of 2.5/1 and 4/1 induced 30% and 39% knockdown in GAPDH protein expression in HeLa cells, respectively. This knockdown is better than siPORT amine-based complexes, which inhibited GAPDH protein expression by only 21% with toxicity, since scrambled siRNA molecules also induced 53% GAPDH reduction compared to untreated cells. “Smart” particles prepared at an N/P ratio of 6/1 also induced 39% knockdown in GAPDH protein expression, which was associated with non-specific toxicity possibly due to the use of excess cationic carrier. To eliminate their toxicity, we decided to use particles prepared at 2.5/1 and 4/1 ratios for the rest of the experiments in HeLa cells. We further utilized qRT-PCR to evaluate the changes in GAPDH mRNA level upon incubation with particles that encapsulated the anti-GAPDH siRNA molecules. As can be seen in Figure 3B, particles prepared at N/P ratios of 2.5/1 and 4/1 induced 40% and 60% knockdown in GAPDH mRNA expression in HeLa cells, respectively, while siPORT amine-based complexes induced 50% knockdown.

In comparison, particles prepared at N/P ratios of 2.5/1 and 4/1 did not induce significant knockdown in GAPDH protein expression in UM-SCC-17B (data not shown), and this may be caused by the delayed acidification of endosomal pH thus causing inefficient cytoplasmic release of siRNA molecules in head and neck cells [21]. Therefore, we evaluated the transfection efficiency of particles prepared at an N/P ratio of 5/1, which induced 14% knockdown in GAPDH protein expression in UM-SCC-17B cells without toxicity while siPORT amine-based complexes induce 36% GAPDH protein knockdown with toxicity (Figure 3C). The low transfection efficiency of “smart” particles in UM-SCC-17B cells is perhaps a result of poor decomplexation of siRNA from the carrier after endosomal escape since a higher amount of polymer was used at an N/P ratio of 5/1 than 2.5/1 and 4/1. Figure 3D shows that particles prepared at 5/1 ratio induced 38% knockdown in GAPDH mRNA expression in UM-SCC-17B cells, compared to siPORT amine-based complexes that induced 54% knockdown. These particles proved to transfect HeLa and UM-SCC-17B cells more effectively than the commercial transfection agent without toxicity.

The observed difference in GAPDH knockdown induced by “smart” particles loaded with anti-GAPDH siRNA in HeLa and UM-SCC-17B cells can be explained by the difference in intracellular pH between these cells lines. The literature shows that normal cells generally have neutral cytosolic (pH 7.2) and acidic endosomal (pH 6.0) and lysosomal (pH 5.0) environment [21,22,23]. Whereas, many tumor cells have an acidified cytosol and more alkaline endosomes/lysosomes with both pH values is around 6.7 [21,22,23]. Although the reason for alkalinization of the endosomal and lysosomal compartments remains elusive, the elevated organelle pH in tumor cells has been confirmed in many reports [21,23,24,25] and proved to dramatically reduce the transfection efficiency of non-viral vectors in tumor cells [26]. Similarly, low GAPDH knockdown in UM-SCC-17B cancer cells can be attributed to endosomal alkalinization, which will reduce the hydrolysis of the hydrazone linkages connecting the membrane-active P(HMA-*co*-TMAEMA) grafts to the polymer backbone. Incomplete release of P(HMA-*co*-TMAEMA) grafts will reduce the net disruption of the endosomal membrane, which will limit the delivery of the loaded anti-GAPDH siRNA into the cytoplasm and diminish the associated GAPDH knockdown. This explains lower GAPDH knockdown observed in UM-SCC-17B cancer cells compared to that observed with HeLa cells. Nevertheless, our results collectively show that “smart” comb-like polymers can function as an effective carrier for enhancing the cytoplasmic delivery of siRNA molecules.

### 2.4. Effect of “Smart” Particles on Bcl-2 Expression

**Figure 4:**
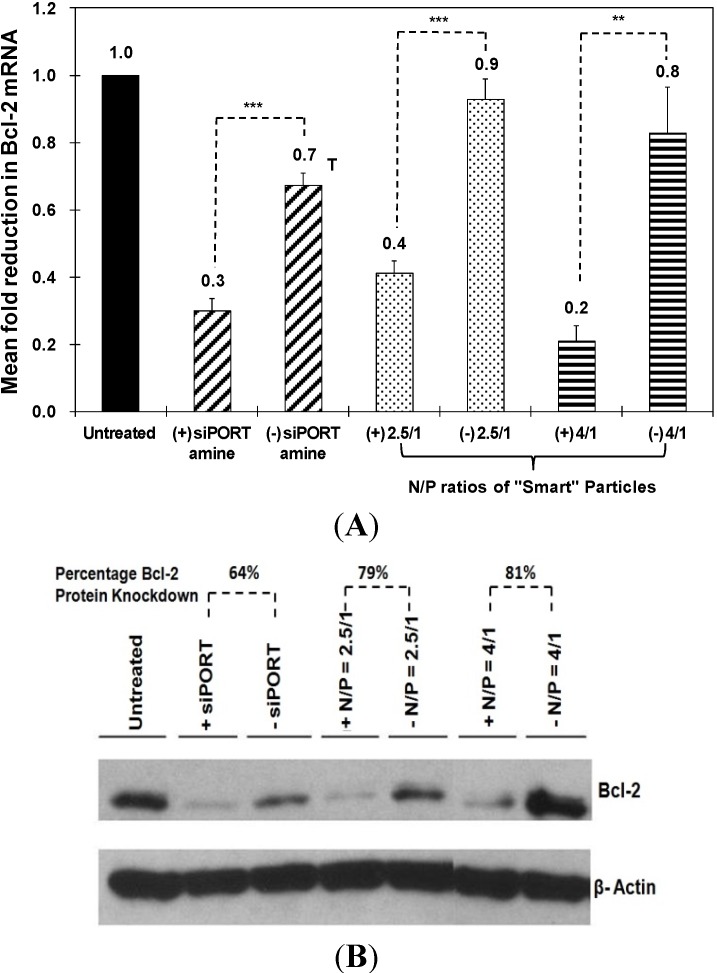
Effect of siPORT amine-based complexes and “smart” nanoparticles prepared by complexation of P(EAA-*co*-BMA)-*b*-PNASI-*g*-P(HMA-*co*-TMAEMA) comb-like polymer with 1.14 µg of the anti-Bcl-2 siRNA (+) or scrambled siRNA (−) at N/P (+/−) ratios of 2.5/1 and 4/1 on Bcl-2 mRNA (**A**) and protein (**B**) levels after treatment for 48 h in HeLa cervical cancer cells. Levels for Bcl-2 mRNA are normalized to the levels of 18S rRNA. Results are the average + the standard error of the mean of five replicates. Statistical difference between particles encapsulating anti-Bcl-2 siRNA (+) and scrambled siRNA (−) was evaluated using paired *t* test where ** denotes *p* ≤ 0.01 and *** denotes *p* ≤ 0.005. Levels for Bcl-2 protein are quantified by Image J software (NIH, Bethesda, MD, USA) and normalized to the levels of β-actin.

**Figure 5:**
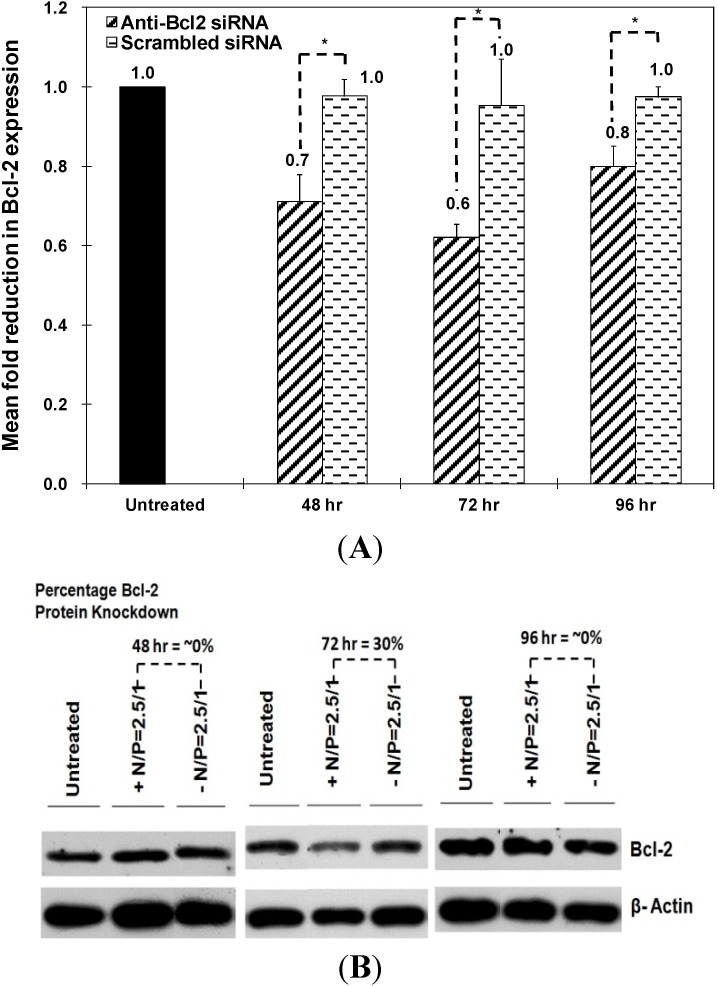
Effect of “smart” nanoparticles prepared by complexation of P(EAA-*co*-BMA)-*b*-PNASI-*g*-P(HMA-*co*-TMAEMA) comb-like polymer with 1.14 µg of the anti-Bcl-2 siRNA (+) or scrambled siRNA (−) at an N/P (+/−) ratio of 2.5/1 on Bcl-2 mRNA (**A**) and protein (**B**) levels at 48, 72, and 96 h in UM-SCC-17B head and neck cancer cells. Levels for Bcl-2 mRNA are normalized to the levels of 18S rRNA. Results are the average + the standard error of the mean of five replicates. Statistical difference between particles encapsulating anti-Bcl-2 siRNA (+) and scrambled siRNA (−) was evaluated using paired *t* test where * denotes *p* ≤ 0.05. Levels for Bcl-2 protein are quantified by Image J software (NIH, Bethesda, MD, USA) and normalized to the levels of β-actin.

The therapeutic activity of “smart” particles was evaluated based on their ability to selectively knockdown Bcl-2 gene expression at both the mRNA and protein levels. We utilized qRT-PCR to measure the changes in Bcl-2 mRNA level upon incubation with particles that encapsulate the anti-Bcl-2 siRNA and compare to those encapsulating a scrambled siRNA sequence. We utilized siPORT amine-based complexes encapsulating an equal dose of anti-Bcl-2 siRNA as a positive control to determine the maximum level of knockdown that can be achieved using robust commercial transfection agents. As shown in Figure 4A, particles prepared at N/P ratios of 2.5/1 and 4/1 selectively induced 50% and 60% knockdown in Bcl-2 mRNA expression in HeLa cells, respectively. This knockdown is better than siPORT amine-based complexes which only inhibited Bcl-2 mRNA expression by 40% accompanied with toxicity. In Figure 4B, particles prepared at N/P ratios of 2.5/1 and 4/1 induced 79% and 81% knockdown in Bcl-2 protein expression in HeLa cells, respectively, while siPORT amine-based complexes induced only 64% knockdown. For UM-SCC-17B cells, in order to exclude the issue of poor decomplexation of particles prepared at an N/P ratio of 5/1, we decreased the N/P ratio to 2.5/1 but increased the incubation time to solve the possible problem of delayed endosomal pH drop. As shown in Figure 5A, Bcl-2 mRNA expression was inhibited by 30%, 40%, and 20% after treatment with particles for 48, 72, and 96 h, respectively. Inhibition of Bcl-2 protein expression was only shown after treatment for 72 h by 30% knockdown (Figure 5B). The results suggested that the therapeutic effects of anti-Bcl-2 siRNA delivered by using comb-like polymer is most effective after treatment for 72 h in UM-SCC-17B cancer cells, which matches earlier studies [27] and suggests the transfection condition should be optimized in different cell types. In summary, these results prove our “smart” comb-like polymer can be utilized as effective carriers for the delivery of therapeutic siRNA molecules into multiple mammalian epithelial cells.

## 3. Experimental Section

### 3.1. Materials

Copper (I) bromide (Cu(I)Br), 2,2'-Azo-bis(isobutyronitrile) (AIBN), butyl methacrylate (BMA), hexyl methacrylate (HMA), *N*-acryloxy succinimide (NASI), *N*,*N*,*N*-trimethyl aminoethyl methacrylate (TMAEMA) and all solvents were purchased from Sigma-Aldrich Chemical Co. (St. Louis, MO, USA). All reagents were used as delivered without further purification except for AIBN, which was crystallized from methanol prior to use. Hexamethyltriethylenetetramine (HMTETA) as a ligand (Aldrich, 97%) was distilled before use. Ethyl acrylic acid (EAA) monomer and 2-dodecylsulfanylthiocarbonylsulfanyl-2-methyl propionic acid (DMP) chain transfer agent were synthesized following published procedures [28,29]. Human anti-GAPDH siRNA, FAM-labeled anti-GAPDH siRNA, negative siRNA sequence, KDalert GAPDH assay kit, and siPORT-NH2 transfection reagent were purchased from Ambion Inc. (Austin, TX, USA). The anti-Bcl-2 siRNA sequence (5'-GCCCUGAUUGUGUAUAUUCA-3') was synthesized by Integrated DNA Technologies, Inc. (Coralville, IA, USA). The RNeasy Mini Kit and Omniscript reverse transcriptase kit were purchased from Qiagen (Valencia, CA, USA). The TaqMan universal PCR master mix and TaqMan gene expression assays for human GAPDH, human Bcl-2, β-actin, and 18S rRNA genes were purchased from Applied Biosystems (Foster, CA, USA). The anti-human β-actin monoclonal antibody and anti-human Bcl-2 monoclonal antibody were purchased from Santa Cruz Biotechnology (Santa Cruz, CA, USA) and BD Biosciences (San Jose, CA, USA), respectively.

### 3.2. Synthesis of pH-Sensitive Comb-Like Polymer

P(EAA-*co*-BMA)-*b*-PNASI-*g*-P(HMA-*co*-TMAEMA) comb-like polymer was synthesized following our established protocol [11]. Basically, the first block of the polymer backbone was synthesized by reversible addition-fragmentation chain transfer (RAFT) polymerization (Scheme IIA). We mixed EAA monomers (1.0 g, 10 mmol) with BMA monomers (0.47 g, 3.3 mmol), DMP (33 mg, 9.15 × 10^−2^ mmol), and AIBN (3 mg, 1.83 × 10^−2^ mmol) in a 50 mL round bottom Schlenk tube. The reaction mixture was degassed by purging with nitrogen for 20 min and placed in an oil bath at 60 °C for 17 h. The resulting crude polymer was dissolved in dimethyl formamide (DMF), precipitated in diethyl ether, and dried under vacuum to yield pure P(EAA-*co*-BMA) polymer. Then P(EAA-*co*-BMA) polymer, was dissolved in dioxane and mixed with N-acryloxy succinimide (NASI) monomers at a 1:56 molar ratio in a round bottom Schlenk tube followed by purging with nitrogen for 15 min. The AIBN initiator (5 mg, 3.0 × 10^−2^ mmol) was added to the reaction mixture before placing the tube in an oil bath at 65 °C for 24 h (Scheme IIB). The crude polymer was dissolved in DMF, precipitated in diethyl ether, and dried under vacuum to yield pure P(EAA-*co*-BMA)-*b*-PNASI copolymer.

**Scheme II:**
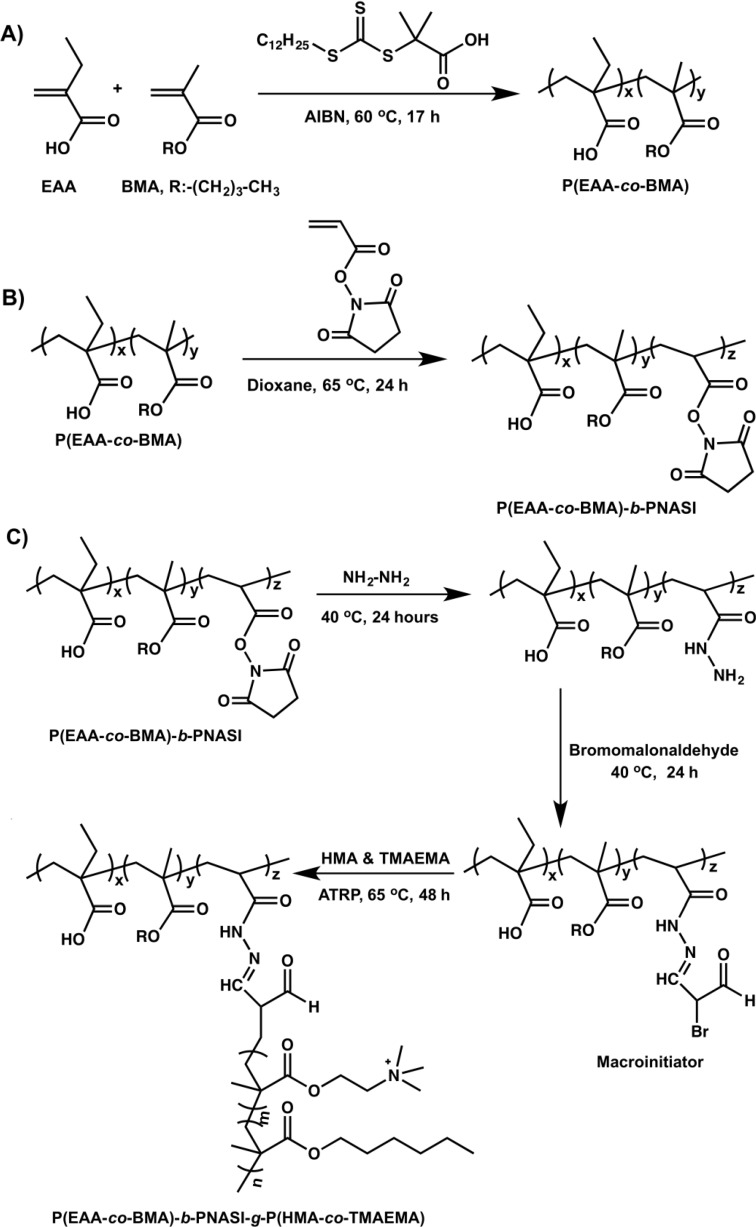
Scheme for synthesis of P(EAA-*co*-BMA)-*b*-PNASI-*g*-P(HMA-*co*-TMAEMA) comb-like polymer.

The diblock polymer backbone with NASI blocks was used to synthesize macroinitiators for graft polymerization of HMA and TMAEMA monomers (Scheme IIC). The polymer was dissolved in dimethyl sulfoxide (DMSO), mixed with anhydrous hydrazine in a flask, and allowed to react at 40 °C for 24 h. The crude product was dissolved in DMF, precipitated in diethyl ether, and dried under vacuum to yield pure polymer-hydrazine conjugates, which was dissolved in DMSO and allowed to react with bromomalonaldehyde at a hydrazine-to-bromomalonaldehyde molar ratio of 1:1.5 for 24 h at room temperature. The pure macroinitiator was precipitated in acetone, filtered, and dried overnight under vacuum. The selected macroinitiator was dissolved in DMF and equimolar concentrations of HMA and TMAEMA monomers were added to the solution. 1/1 molar ratio of [Cu(I)Br]/HMTETA ligand addition followed by three freeze-vacuum-thaw cycles before placing the reaction mixture in an oil bath at 60 °C for 48 h while stirring. The molar ratio of the macroinitiator, HMA, and TMAEMA were controlled to prepare P(HMA-*co*-TMAEMA) grafts with a number average molecular weight (Mn) of 20 KDa equally split between the HMA and TMAEMA units. The final product P(EAA-*co*-BMA)-*b*-PNASI-*g*-P(HMA-*co*-TMAEMA) comb-like polymer was precipitated in diethyl ether, dried under vacuum, and further purified by dialysis against NaOH solution (pH = 10) for 24 h followed by lyophilization for 48 h. Figure S1 shows the ^1^H-NMR spectra of P(EAA-*co*-BMA), P(EAA-*co*-BMA)-*b*-PNASI, and P(EAA-*co*-BMA)-*b*-PNASI-*g*-P(HMA-*co*-TMAEMA) copolymers.

### 3.3. Formulation and Characterization of “Smart” Particles

P(EAA-*co*-BMA)-*b*-PNASI-*g*-P(HMA-*co*-TMAEMA) comb-like polymer was dissolved in RNase-free water and mixed with 0.7 μg of anti-Bcl-2 siRNA molecules dissolved in 1 μL of RNase-free water at different nitrogen/phosphate (N/P) ratios. Each mixture was vortexed and allowed to stand at room temperature for 20 min before loading onto a 1% w/V agarose gel containing ethidium bromide (EtBr). The gel was immersed in a Tris-acetate-EDTA (TAE) buffer and run at 60 V for 45 min and visualized under UV (Fotodyne Incorporated, Hartland, WI, USA). Size and zeta potential of the particles prepared at N/P ratios of 2.5/1, 4/1, and 5/1 were measured using 90Plus particle size analyzer with ZetaPALS capability (Brookhaven Instruments Corporation, Holtsville, NY, USA).

### 3.4. Culture of HeLa and UM-SCC-17B Cells

HeLa cervical cancer and UM-SCC-17B head and neck cancer cells were generously provided by Nör and cultured following established protocols. Briefly, HeLa and UM-SCC-17B cells were maintained in DMEM supplemented with 10% fetal bovine serum, 10,000 units/mL penicillin, 10,000 μg/mL streptomycin and regularly changing the growth medium every 2 days. Cells were incubated at 37 °C, 5% CO_2_, 95% relative humidity, and passaged upon reaching 70%–90% confluency using 0.25% trypsin/EDTA mixture.

### 3.5. Cellular Uptake of “Smart” Particles

Comb-like polymer and commercial siPORT-NH2 were dissolved in OPTI-MEM solution and mixed with 0.57 µg of FAM-labeled anti-GAPDH siRNA molecules at N/P ratios of 1.5/1, 2.5/1, 4/1, 8/1, and 12/1 to prepare different particles that were incubated with HeLa and UM-SCC-17B cells for 6 h at 37 °C, 5% CO_2_ and 95% relative humidity. HeLa and UM-SCC-17B cells were washed with PBS, treated with 0.25% trypsin/EDTA solution for 10 min, harvested, and centrifuged to remove the supernatant and form a cell pellet. Cell pellets were suspended in PBS and analyzed using Biosciences FACSCalibur (Becton Dickinson, Franklin Lakes, NJ, USA) to determine the percentage of fluorescently-labeled HeLa and UM-SCC-17B cells for each treatment. HeLa and UM-SCC-17B cells were gated by forward/side scatter and 10,000 gated events were collected per sample to discriminate between live and dead cells and account for live cells only.

### 3.6. In Vitro Evaluation of GAPDH Knockdown in HeLa and UM-SCC-17B Cells

HeLa and UM-SCC-17B cells were plated in 24-well plates at a seeding density of 20,000 cells/well and allowed to adhere for 18 h. The “smart” particles and siPORT-NH2 complexes incorporating 1.14 µg of anti-GAPDH siRNA or control siRNA molecules were incubated with HeLa and UM-SCC-17B cells at a final siRNA concentration of 200 nM for 6 h followed by addition of 500 μL of fresh culture medium and incubation for a total of 48 h. The effect of different treatments on GAPDH expression was quantified based on mRNA and protein levels. The amount of GAPDH protein expressed by HeLa and UM-SCC-17B cancer cells was measured using the KDalert GAPDH assay following manufacturer’s specifications. The level of GAPDH protein expression in response to different treatments was normalized to that of untreated control cells. For quantification of GAPDH mRNA, total RNA was isolated from HeLa and UM-SCC-17B cells using the RNeasy Mini Kit and 0.25 μg of total RNA was reverse transcribed using Omniscript reverse transcriptase kit following manufacturer’s protocols. Real-time PCR was performed in a final volume of 20 μL containing 2 μL of cDNA (corresponding to 10 ng of total RNA for GAPDH and β-actin amplification), 1 μL of each primer, and 10 μL of the qPCR MasterMix in the 7500 Fast Real-Time PCR system.

### 3.7. In Vitro Evaluation of Bcl-2 Knockdown in HeLa and UM-SCC-17B Cells

HeLa and UM-SCC-17B cells were plated in 24-well plates at a seeding density of 20,000 cells/well and allowed to adhere for 18 h. The “smart” particles and siPORT-NH2 complexes incorporating 1.14 µg of anti-Bcl-2 siRNA or control siRNA molecules were incubated with HeLa and UM-SCC-17B cells at a final siRNA concentration of 200 nM for 6 h followed by addition of 500 μL of fresh culture medium and incubation for a total of 48, 72, or 96 h. The amount of Bcl-2 protein expressed by HeLa and UM-SCC-17B cells was analyzed using the western blot technique following established protocol [30]. Briefly, whole cell lysates were resolved by SDS-PAGE and membranes were probed overnight at 4 °C with anti-human β-actin monoclonal antibody (1:1,000,000) and anti-human Bcl-2 monoclonal antibody (1:1000), and proteins were visualized with SuperSignal West Pico Chemiluminescent Substrate (Pierce, Rockford, IL, USA). The knockdown of Bcl-2 protein expression in response to different treatments was quantified by Image J software (NIH, Bethesda, MD, USA) and normalized to that of untreated cells. For quantification of mRNA, total RNA was isolated from HeLa and UM-SCC-17B cells using the RNeasy Mini Kit and 0.25 μg of total RNA was reverse transcribed using Omniscript reverse transcriptase kit following manufacturer’s protocols. Real-time PCR was performed in a final volume of 20 μL containing 2 μL of cDNA (corresponding to 10 ng of total RNA for Bcl-2 and 18S rRNA amplification), 1 μL of each primer, and 10 μL of the qPCR MasterMix in the 7500 Fast Real-Time PCR system.

## 4. Conclusions

In summary, we proved that our “smart” pH-sensitive, membrane-destabilizing, comb-like polymer could successfully complex model siRNA molecules into stable nanoparticles at low N/P ratios, which indicates their ability to encapsulate large doses of therapeutic nucleic acids with minimum toxicity. These particles proved to be efficiently internalized by cancer cells and selectively knockdown GAPDH and Bcl-2 expression at both the protein and mRNA levels. The results collectively indicate the potential of these particles to serve as a carrier for silencing Bcl-2 expression in cancer cells.

## Supplementary Files

Supplementary File 1
