# Supplementary material for: Silencing Bcl-2 Expression in Epithelial Cancer Cells Using “Smart” Particles
Source: J Funct Biomater. 2014 Sep 16;5(3):167–82. doi: 10.3390/jfb5030167 (PMC4192611; doi:10.3390/jfb5030167)
Supplement: Supplementary File 1 [file jfb-05-00167-s001.pdf]

J. Funct. Biomater. **2014**, *5*

OPEN ACCESS
Journal of
Functional Biomaterials
ISSN 2079-4983
www.mdpi.com/journal/jfb

Supplementary Information

Figure S1. (**A**) The ^{1}H-NMR spectrum of P(EAA-*co*-BMA) copolymer in DMSO-d6; (**B**) the ^{1}H-NMR spectrum of P(EAA-*co*-BMA)-*b*-PNASI copolymer in DMSO-d6; (**C**) the ^{1}H-NMR spectrum of P(EAA-*co*-BMA)-*b*-PNASI-*g*-P(HMA-*co*-TMAEMA) comb-like polymer in D_2O.

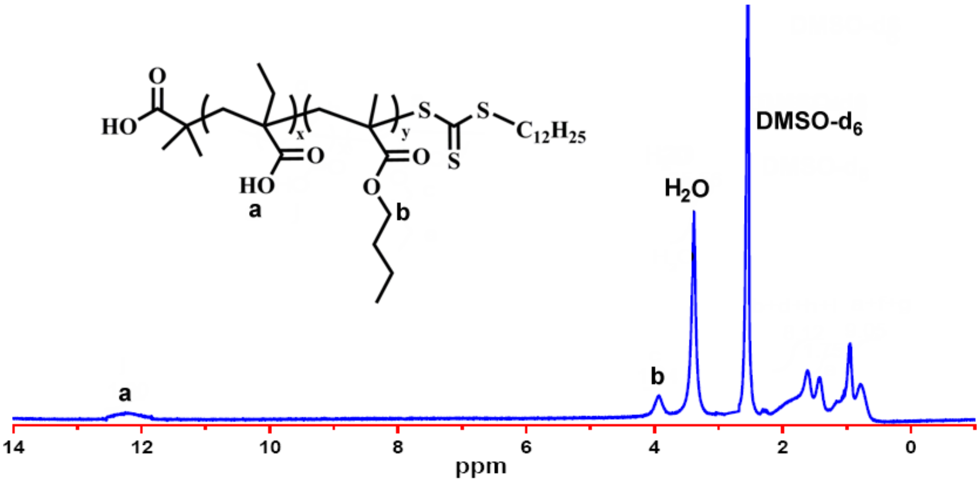

(**A**)

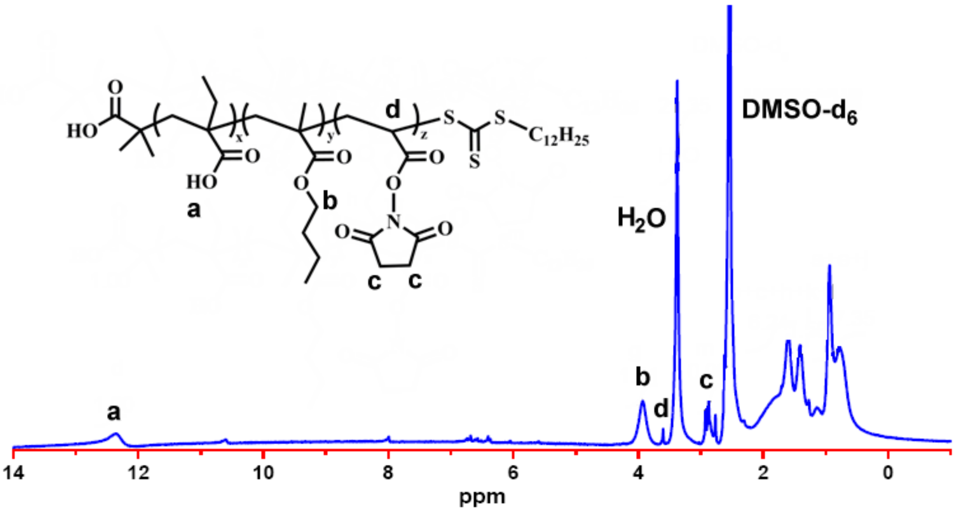

(**B**)

Figure S1. *Cont.*

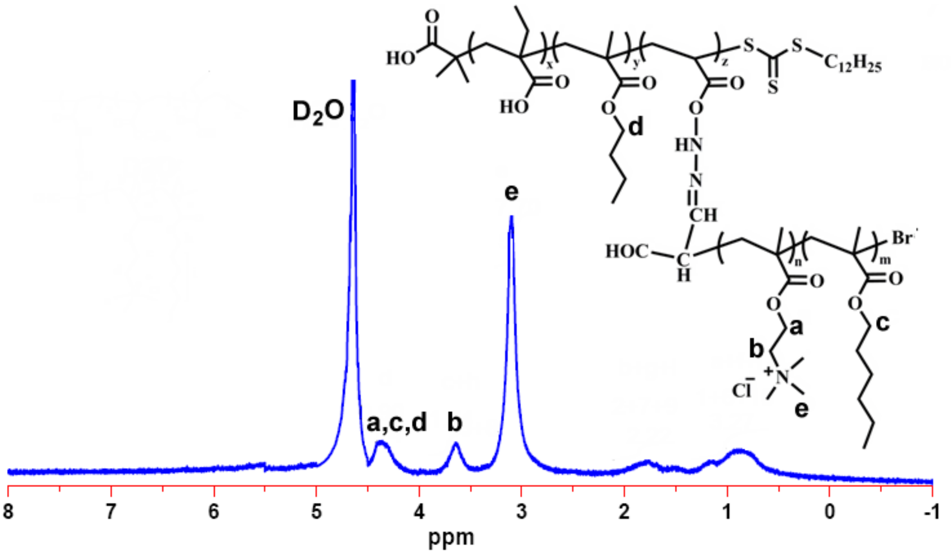

(C)

© 2014 by the authors; licensee MDPI, Basel, Switzerland. This article is an open access article distributed under the terms and conditions of the Creative Commons Attribution license (http://creativecommons.org/licenses/by/3.0/).
